# Como o Exame Físico Cardiovascular Impacta a Tomada de Decisão Clínica em Vários Cenários de Doenças Valvulares Cardíacas

**DOI:** 10.36660/abc.20240272

**Published:** 2025-03-06

**Authors:** Isabella Silveira Teixeira, Vinícius Lima Borges, Natan Viola, Henrique Turin Moreira, Antonio Pazin, André Schmidt, José Antônio Marin-Neto, Minna Moreira Dias Romano

**Affiliations:** 1 Faculdade de Medicina de Ribeirão Preto Universidade de São Paulo Ribeirão Preto SP Brasil Faculdade de Medicina de Ribeirão Preto da Universidade de São Paulo, Ribeirão Preto, SP – Brasil

**Keywords:** Exame Físico, Ecocardiografia, Tomada de Decisão Clínica, Doenças das Valvas Cardíacas

## Abstract

**Fundamento:**

Os exames complementares de diagnóstico substituíram a importância do exame físico (EF) na tomada de decisão clínica (TDC).

**Objetivo:**

Avaliar o impacto do EF cardiovascular em cenários de doenças valvulares cardíacas.

**Método:**

Estudo intervencionista realizado com participantes voluntários que tiveram ou não a oportunidade de realizar EF em cenários de valvopatias. O EF foi simulado em um simulador cardiopulmonar de alta fidelidade. Os participantes receberam perguntas sobre a TDC antes e depois de receberem um laudo ECO Concordante ou ECO Discordante. O coeficiente kappa de Cohen e as tabelas de contingência quadradas compararam a precisão diagnóstica. Os testes ANOVA compararam o número de exames solicitados; o nível de significância foi estabelecido em p < 0,05.

**Resultados:**

Sessenta participantes realizaram 239 observações clínicas em 4 disfunções valvulares. A acurácia diagnóstica da disfunção valvular foi boa (kappa = 0,935, p < 0,001). Após receber os laudos do ECO, a precisão foi pior sem EF (p = 0,0047). O nível de confiança no diagnóstico foi 28,18% maior quando o EF foi realizado (p < 0,01). Entretanto, após receber os laudos do ECO, os níveis de confiança diagnóstica foram apenas 4% maiores no grupo com EF (p = 0,03). Houve significativamente mais solicitações de cateterismo cardíaco (CATE) quando o EF não foi realizado (p = 0,0326). A indicação de intervenção valvular não foi relacionada à oportunidade ou não de realizar EF (79 com EF vs. 78 sem EF, p = 0,0607), mas foi influenciada pelos laudos do ECO Concordante vs. ECO Discordante (p < 0,001).

**Conclusões:**

A realização de EF aumentou a confiança no diagnóstico em cenários de valvopatia e a classificação correta da disfunção. As decisões de tratamento foram mais baseadas nos laudos do ECO do que no EF quando eram discordantes, e as solicitações de CATE aumentaram quando os participantes não tiveram a oportunidade de realizar EF.

## Mensagens-Chave

1.A prática clínica médica perdeu a confiança no exame físico (EF) em comparação aos exames complementares (EC) de diagnóstico. Mais importância tem sido dada ao ecocardiograma (ECO) em cenários de disfunção valvular cardíaca. Nenhum estudo mediu o impacto do EF cardíaco na tomada de decisão clínica (TDC).

2.Discrepâncias entre os resultados do EF e os laudos do ECO são comuns e podem impactar a TDC de diferentes formas.

3.O EF provou ser essencial para aumentar a confiança no diagnóstico e a classificação de disfunções valvulares cardíacas. O ECO ainda desempenha um papel significativo na TDC, mesmo quando discordante do EF.

4.O número de solicitações de um exame invasivo, como o cateterismo cardíaco (CATE), é influenciado pelo EF. O número de solicitações foi maior quando os participantes não realizaram EF.

## Graphical abstract

**Figura Central:**
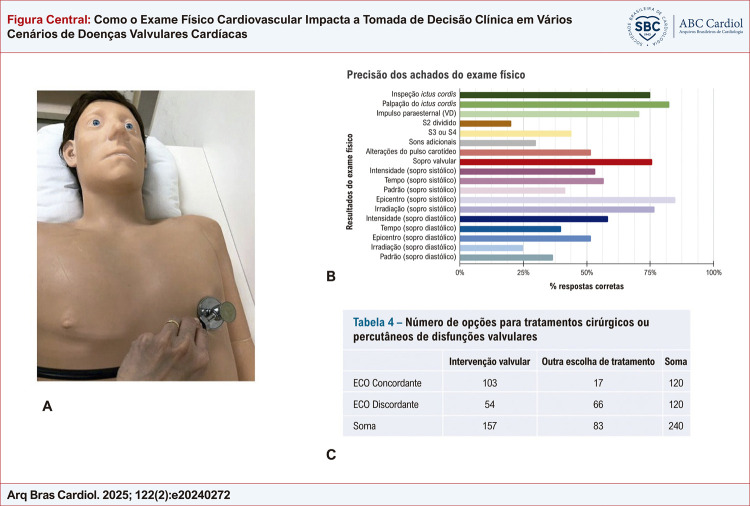
: Como o Exame Físico Cardiovascular Impacta a Tomada de Decisão Clínica em Vários Cenários de Doenças Valvulares Cardíacas

Harvey – Simulador cardiopulmonar. (A) Precisão dos achados do exame físico em todos os tipos de doenças valvulares cardíacas; (B) Número de opções de tratamento valvular após receber um ecocardiograma discordante (C).

## Introdução

Após honrada reputação há séculos, nas últimas décadas, a importância do EF para a TDC perdeu muito de seu apelo, e uma clara tendência surgiu para sua substituição por vários EC de diagnóstico.^1^ Na cardiologia, surgiu uma preocupação genuína sobre a real importância do EF desde o surgimento de tantos exames de diagnóstico por imagem, entre os quais o mais disponível é o ECO transtorácico. Entretanto, recorrer a um EC não precedido por um EF adequado pode aumentar os custos e o tempo de diagnóstico e potencialmente causar distorções graves, incluindo erros de prognóstico.^2^ Outra consequência previsível dessa tendência é que os profissionais podem ter perdido a capacidade clínica de detectar doenças cardíacas, como valvopatias,^1 , 3^ impactando severamente a confiança dos profissionais no EF cardiovascular. Além disso, embora o ensino sobre o EF cardiovascular tenha sido aperfeiçoado com o uso de simuladores de alto desempenho, há evidências claras de que as habilidades educacionais para ensinar EF cardíaco foram colocadas em segundo plano no currículo de muitos cursos médicos.^3 - 7^

Há grande conhecimento sobre o valor do EF cardiovascular na detecção de anormalidades cardiovasculares, como doenças valvulares, bem como elevação das pressões cardíacas, arritmias, dilatação das câmaras e disfunção sistólica e diastólica.^1 , 8^ A maioria dos estudos publicados comparou a concordância entre os sinais físicos cardíacos e outros métodos de diagnóstico, como o ECO^1^ ou CATE, por meio de medições da pressão intracardíaca e outros índices de estrutura e função anormais do coração. Entretanto, nenhum estudo anterior havia testado o papel do EF na TDC, particularmente quando se considera o impacto de um EC mais sofisticado, porém acessível, como o ECO.^9^

A TDC envolve habilidades técnicas e fatores complexos e “difíceis de mensurar”, como pensamento cognitivo do tipo analítico individual ou de “reconhecimento de padrões”.^10 , 11^ Estudos anteriores, como os de Sibbald et al., estabeleceram o papel da anamnese como uma ferramenta para melhorar o diagnóstico em valvopatias simuladas, onde o EF foi estruturado e simulado em sistemas cardiopulmonares de alta fidelidade.^12^ Hampton et al. avaliaram a importância relativa da anamnese combinada ao EF e aos exames laboratoriais na TDC em alguns cenários clínicos gerais, mas não especificamente em valvopatias. Em um grupo de 80 pacientes com doenças cardíacas, a anamnese influenciou positivamente a obtenção do diagnóstico em 80% das situações estudadas. Entretanto, apenas em algumas proporções de pacientes, o diagnóstico foi melhorado quando o EF foi adicionado à anamnese.^13^ Embora o EF também possa ter tido um papel significativo nas decisões prognósticas e terapêuticas, esses aspectos não foram avaliados exaustivamente nesses estudos.

Buscamos avaliar o impacto do EF cardiovascular na TDC de estudantes de graduação em medicina em alguns cenários de doenças valvulares cardíacas graves, simulados com um simulador cardiopulmonar de alta fidelidade.^10 , 14^

## Métodos

O estudo foi desenhado como intervencionista, no qual a variável de intervenção foi o EF cardiovascular, e as variáveis dependentes (desfechos) foram as respostas que refletiam a TDC. Foi aprovado pelo Comitê Institucional de Ética em Pesquisa (processo número HC-FMRP-5.046.039).

### População

Estudantes de graduação em medicina da Faculdade de Medicina de Ribeirão Preto da Universidade de São Paulo, cursando o 4º, 5º ou 6º ano de graduação, foram convidados a participar. O tamanho da amostra de conveniência foi baseado em 200 alunos disponíveis no momento do estudo. Os alunos receberam convites e instruções por e-mail institucional e grupos em redes sociais. Os participantes deveriam ser alunos previamente aprovados na disciplina cardiovascular de seu curso clínico (4º ano acadêmico), e a participação era totalmente voluntária. Todos os alunos que participaram forneceram consentimento por escrito.

### Simulação

O EF foi realizado em um simulador cardiopulmonar de alta fidelidade (Harvey^@^, *Gordon Center for Research in Medical Education, University of Miami* , FL, EUA), capaz de reproduzir sinais físicos normais e anormais de diversas condições cardiovasculares. Embora todo o grupo de alunos já tivesse adquirido experiência com o simulador Harvey durante a disciplina cardiovascular, todos eles receberam treinamento padronizado adicional no uso do simulador Harvey com vários exemplos de condições simuladas após se voluntariarem para participar.

### Desenho

Uma simulação de quatro cenários de doença valvular (estenose mitral (EM), regurgitação mitral (RM), estenose aórtica (EA) e regurgitação aórtica (RA)) foi elaborada por dois cardiologistas experientes. Cada cenário começou com uma breve sinopse do caso clínico ( Apêndice 1 ), que forneceu informações sobre um paciente fictício (idade, sexo, breve caracterização de sintomas como dispneia, angina, edema, palpitação, síncope e menção de um antecedente individual de um sopro cardíaco não especificado). Após a leitura da anamnese e sinopse de cada cenário, os participantes foram divididos aleatoriamente em grupos com ou sem a oportunidade de realizar EF cardiovascular no simulador e, em seguida, receberam um laudo de ECO por escrito. Foi um processo simples de randomização baseado em tabelas geradas por computador dentro do software estatístico. Todos os cenários de doença valvular foram planejados como típicos, com sintomas aparentes e simulação de EF cardiovascular, que incluía sinais de alta gravidade.

Um ecocardiografista experiente desenvolveu os laudos do ECO. Para cada um dos cenários valvulares foram elaborados dois laudos diferentes: um laudo concordante (ECO Concordante) e um laudo discordante (ECO Discordante) ( Apêndice 2 ). O laudo concordante apresentou evidências de valvopatia grave concordante com sintomas e EF. Mostrou medidas como área valvular < 1 cm^2^ em EA ou mitral, gradientes transvalvulares médios elevados e quantificações de RA ou mitral grave. O laudo discordante (ECO Discordante) continha informações inconsistentes (área valvular ou gradientes não fornecidos, informações anatômicas ausentes ou nenhuma divulgação da gravidade da regurgitação, que foi classificada apenas como uma doença valvular leve).

**Figura 1:**
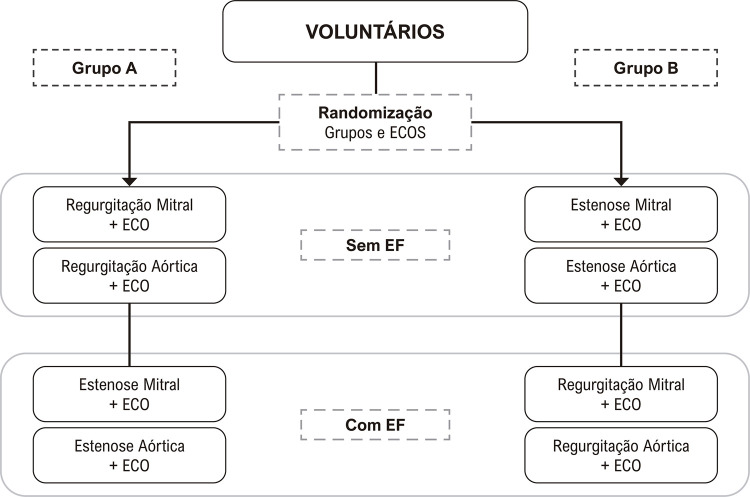
– Distribuição dos cenários valvulares nos grupos com e sem possibilidade de realização de exame físico (EF). ECO: ecocardiograma.

A aplicação de cenários aos participantes foi conduzida como um exame clínico objetivo estruturado formativo (OSCE) ( Apêndice 3 ). Cada participante foi exposto aos quatro casos de cenários de valvopatia, EM, RM, EA e RA, em ordem de sequência aleatória (Grupos A e B) ( Figura 1 ). Para randomizar a chance de realizar EF, a variável intervencionista, os participantes foram selecionados aleatoriamente para ter a oportunidade de realizar EF em metade dos cenários. Após esta etapa, os participantes receberam o laudo do ECO de forma aleatória, sendo ECO Concordante ou ECO Discordante no caso clínico simulado.

Perguntas sobre a TDC foram aplicadas aos participantes após receberem anamnese e terem ou não tido a oportunidade de realizar EF cardiovascular. Em uma etapa subsequente, os participantes receberam laudos de ECO concordante ou discordante. A duração média do OSCE foi de 20 minutos.

### Desfechos

A TDC foi avaliada como respostas a questões relacionadas ao diagnóstico clínico, etiologia das doenças valvulares cardíacas, nível de confiança no diagnóstico, tipos de EC para diagnóstico e decisões de tratamento ( Apêndice 3 ). Perguntas relacionadas ao diagnóstico foram apresentadas após a não realização de EF cardiovascular e novamente após a leitura dos laudos ECO Concordante ou ECO Discordante. As questões sobre o diagnóstico incluíram se a hipótese de “patologia valvular” era provável, sua gravidade, o nível de confiança no diagnóstico e a seleção dos exames EC. Perguntas relacionadas às decisões de tratamento foram apresentadas somente após a leitura dos laudos do ECO. Nesse momento, os participantes foram questionados novamente sobre o tipo de patologia valvular, sua gravidade, seu nível de confiança no diagnóstico e a decisão terapêutica. Por definição, metade dos participantes foi obrigada a responder perguntas sobre a TDC, mesmo em cenários nos quais não tiveram a oportunidade de realizar EF cardíaco, confiando apenas na anamnese e nas informações do laudo do ECO. O nível de confiança na hipótese diagnóstica antes e após a interpretação do ECO foi classificado em uma escala Likert^15^ de 6 pontos e codificado como média ± DP.

### Avaliação da qualidade do exame físico

O desempenho de cada participante na detecção de anormalidades durante o EF usando o simulador Harvey foi avaliado por um dos pesquisadores com uma lista de verificação OSCE ( Apêndice 3 ), adaptada de listas de verificação de EF cardiovascular validadas anteriormente.^10 , 16^ A inspeção completa da região precordial, palpação do impulso do ventrículo direito e do ápice cardíaco, ausculta dos sons cardíacos (S3, S4, sopros diastólicos e sistólicos, cliques e estalos de abertura) e caracterização do desenho dos pulsos arteriais carotídeos (se regulares, *parvus-tardus* ou pulso de Corrigan) foram todos incluídos na lista de verificação. Os participantes tiveram dez minutos para completar o EF. Antes de prosseguir para o questionário e interpretação do ECO, eles foram solicitados a indicar ao examinador todos os achados normais ou anormais daquele paciente simulado. Os participantes foram autorizados a reexaminar o simulador em caso de dúvida. A presença ou ausência de achados foram codificadas como variáveis binárias. Cada participante respondeu ao questionário de forma digital em seus smartphones pessoais.

### Descrição dos dados e análise estatística

A qualidade do EF foi expressa em porcentagens de respostas corretas em comparação com um modelo dos manuais do simulador Harvey. Respostas corretas foram pontuadas quando participantes indicaram sinais no EF que estavam presentes no modelo do fabricante. As respostas às perguntas sobre a TDC foram coletadas de todos os participantes em uma única planilha (Excel). As variáveis categóricas foram expressas como porcentagens e frequências, e as variáveis contínuas foram expressas como médias e desvios padrão. O nível de confiança no diagnóstico pré e pós-interpretação do ECO foi comparado usando o teste t de Student não pareado, conforme apropriado. A normalidade dos dados contínuos foi testada com o teste de Kolmogorov–Smirnov. O coeficiente kappa de Cohen e as tabelas de contingência quadradas foram usados para comparar o resultado da precisão do diagnóstico entre as quatro patologias valvulares antes e após o EF e pré e pós laudos do ECO. Testes ANOVA unidirecionais (sem teste *post hoc* ) foram utilizados para comparar o número de exames diagnósticos solicitados por participantes em diferentes cenários de valvopatias. A identificação de sinais incorretos e respostas omitidas foram interpretadas como erradas. Todas as análises estatísticas foram realizadas usando o software Stata 14.0 (StataCorp, College Station, TX) e o nível de significância foi definido como p < 0,05.

## Resultados

**Figura 2:**
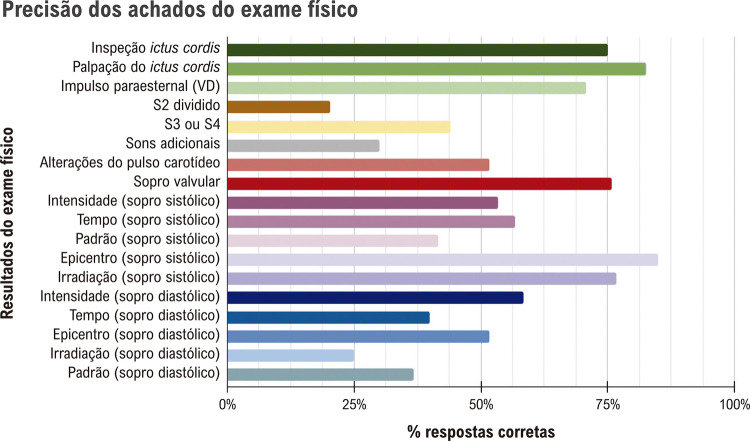
– Qualidade do exame físico considerando todos os casos simulados de quatro disfunções valvulares cardíacas.

A população do estudo foi composta por 60 participantes voluntários que participaram de quatro tipos de cenários de disfunção valvular, resultando em 239 observações, representando uma instância de dados ausentes. Metade dos participantes realizou EF (resultando em 120 casos), e a outra metade não teve a oportunidade de realizar EF (119 casos devido à ausência de um). Vinte e três participantes estavam no 4º ano, 23 no 5º ano e 14 participantes no 6º ano do curso de medicina (6 anos em nossa Faculdade de Medicina). A qualidade geral do EF cardíaco é demonstrada na Figura 2 .

**Figura 3:**
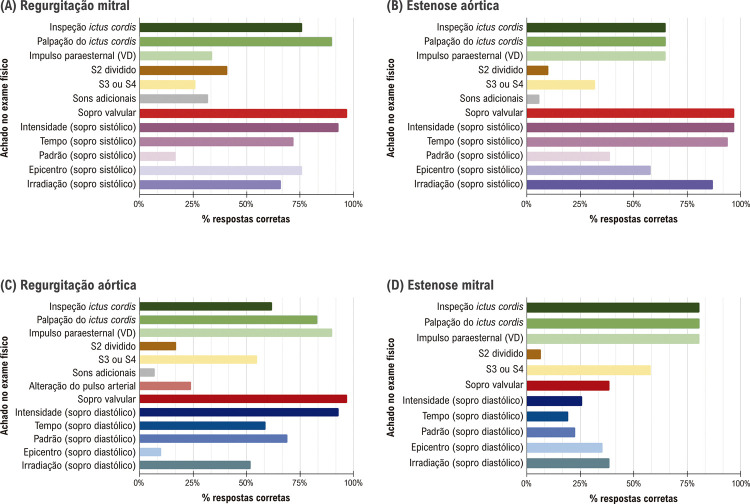
– Qualidade do EF considerando cada disfunção valvular: (A) Regurgitação Mitral, (B) Estenose Aórtica, (C) Regurgitação Aórtica e (D) Estenose Mitral.

Os participantes detectaram corretamente o *ictus cordis* em 75% (n = 90) dos casos. O impulso paraesternal do ventrículo direito foi identificado em 71% dos casos (n = 85). Os sopros cardíacos foram identificados corretamente em mais de 76% dos casos (n = 91), principalmente quando havia sopros sistólicos. As características dos sopros sistólicos como irradiação foram identificadas corretamente em mais de 77% (n = 92) dos casos, mas a porcentagem de respostas corretas foi inferior a 60% (n = 63) para sopros diastólicos. Sons adicionais, como S3 e S4, foram reconhecidos em 44% (n = 53) dos casos. Quando a qualidade do EF foi comparada entre diferentes valvopatias ( Figura 3 ), os sinais de sopros relacionados à sístole, como EA e RM, foram mais facilmente reconhecidos do que os sopros relacionados à diastólica, como EM e RA. O pior desempenho no EF cardíaco foi relacionado à identificação e caracterização do sopro devido à EM.

A capacidade diagnóstica para identificar o tipo de disfunção valvular (EA, RA, RM ou EM) foi boa (kappa = 0,935, p < 0,001, intervalo de confiança [0,798-0,939]), mesmo quando os participantes receberam um laudo discordante do ECO ( Tabela 1 ).

**Tabela 1:** – Concordância entre o diagnóstico de disfunção valvular antes e depois do recebimento dos laudos do ECO

Diagnóstico pré-ECO	Diagnóstico pós-ECO				Soma
	1	2	3	4	
1	55	0	1	4	60
2	1	51	1	1	54
3	0	0	54	0	54
4	3	0	0	55	58
**Soma**	59	51	56	60	226

Treze participantes não responderam. Cenários: 1 = Regurgitação Mitral (RM), 2 = Regurgitação Aórtica (RA), 3 = Estenose Aórtica (EA) e 4 = Estenose Mitral (EM).

Voluntários que não tiveram oportunidade de realizar o EF foram menos propensos a avaliar corretamente o grau de disfunção valvular (a resposta correta) após receberem laudos do ECO Concordante ou ECO Discordante (a resposta correta sobre a gravidade com EF = 85 vs. sem EF = 64; p = 0,0047), (kappa = 0,2887, p < 0,001, IC [0,797-0,931]) ( Tabela 2 ).

**Tabela 2:** – Concordância entre a classificação da disfunção valvular antes e depois do recebimento dos laudos do ECO

Classificação pré-ECO	Classificação pós-ECO			Soma
	1	2	3	
1	40	28	0	68
2	40	109	0	149
3	11	8	3	22
**Soma**	91	140	8	239

1 = leve; 2 = grave; 3 = sem resposta sobre a gravidade da disfunção valvular.

O nível de confiança (escala Likert) no diagnóstico foi 28,18% maior no grupo de participantes que realizaram EF. Quando a confiança no diagnóstico foi confrontada com os laudos do ECO, que poderiam ser ECO Concordante ou ECO Discordante (a principal discordância foi sobre a classificação da valvopatia), os níveis de confiança no diagnóstico aumentaram apenas 4% com a realização do EF ( Tabela 3 ).

**Tabela 3:** – Nível de confiança na disfunção valvular

	Escala de Likert	Significância estatística
Com EF	4,64	p < 0,01
Sem EF	3,62	
Com EF após laudos do ECO	5,24	p = 0,03
Sem EF após laudos do ECO	5,03	

**Figura 4:**
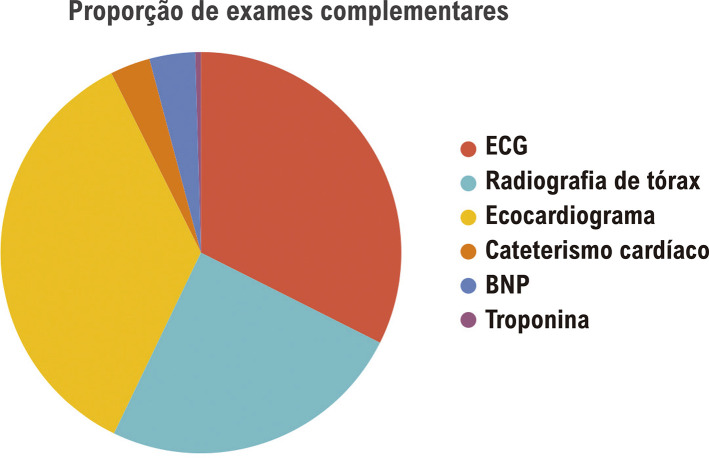
– Proporção de exames complementares solicitados pelos participantes para diagnóstico.

Os participantes solicitaram uma mediana de três EC de diagnóstico; na maioria das vezes, um ECG, uma radiografia de tórax ou um ECO ( Figura 4 ).

O número total de EC de diagnóstico necessários foi de 650, sendo que 328 foram indicados pelo grupo de participantes que realizaram EF, enquanto 322 foram indicados pelo grupo que não realizou EF. O número de indicações de EC foi semelhante entre os quatro cenários de valvopatias (ANOVA p = 0,117 em comparação aos quatro grupos).

Ao considerar a realização de EF, houve um número significativamente maior de EC solicitados pelo grupo sem EF para cenários específicos de casos de RA (74 com EF vs. 103 sem EF; p = 0,0009). Em contraste, mais EC foram solicitados por participantes que realizaram EF nos cenários de casos de EA (93 com EF vs. 69 sem EF; p = 0,0083) e EM (84 com EF vs. 70 sem EF; p = 0,045), e nenhuma diferença significativa foi observada em RM (77 com EF vs. 80 sem EF; p> 0,05). Além disso, considerando todos os cenários de disfunções valvopatológicas, houve um número significativamente maior de CATE solicitados por participantes que não puderam realizar o EF (13 sem EF vs. 8 com EF, p = 0,0326).

A indicação terapêutica de intervenção valvular não foi influenciada pela realização de EF (79 com EF vs. 78 sem EF, p = 0,0607). Entretanto, os participantes que receberam laudos ECO Concordantes escolheram significativamente mais vezes a opção de tratamento cirúrgico das disfunções valvulares (p < 0,001) em comparação com aqueles que receberam laudas ECO Discordantes ( Tabela 4 ).

**Tabela 4:** – Número de escolhas para indicação de intervenções valvulares

	Intervenção valvular	Outra escolha de tratamento	Soma
ECO Concordante	103	17	120
ECO Discordante	54	66	120
Soma	157	83	240

Intervenção valvular: cirúrgica ou percutânea.

## Discussão

Os resultados desta investigação mostraram que, em um grupo de estudantes de medicina que já haviam cursado a disciplina cardiovascular (4º ano acadêmico), o nível de confiança no diagnóstico de disfunções valvulares e na sua gravidade, mesmo após receberem um laudo de ECO, aumentou quando os participantes tiveram a oportunidade de realizar EF. A realização de EF não demonstrou capacidade incremental de detectar o tipo de disfunção valvular simulada após consideração do laudo do ECO. Entretanto, os participantes que conseguiram realizar EF tiveram maior capacidade de não influenciar o grau de disfunção valvular e questionar os laudos de ECO do que o grupo que precisou tomar uma decisão clínica, apenas com base na anamnese e no ECO. A realização de EF também não afetou a indicação de tratamento cirúrgico de nenhuma disfunção valvular. Além disso, receber um laudo ECO Concordante ou ECO Discordante em relação aos cenários clínicos influenciou significativamente o tratamento sugerido para a doença valvular específica, independentemente da realização de EF.

Outro achado relevante do estudo foi que, no geral, o desempenho dos estudantes de medicina no EF foi bom para detectar vários sinais de doenças valvulares. O desempenho foi melhor na detecção de sinais valvulares regurgitantes e EA e pior na detecção de sinais de EM. Ainda, com exceção da solicitação de CATE, não houve aumento nas indicações de EC adicional para fazer o diagnóstico quando o EF não foi realizado. Isso reflete que todos os laudos de ECO, até mesmo o laudo ECO Discordante, mostraram corretamente os sinais da disfunção valvular simulada, e a discordância apresentada estava relacionada à gravidade da doença.

Estudos anteriores avaliaram a precisão de sinais isolados de disfunção valvular no EF para identificar condições patológicas em comparação com outras ferramentas diagnósticas instrumentadas, como o ECO.^1 , 17^ É importante enfatizar que, desconsiderando uma ferramenta simples como o EF, que pode ser realizado com boa precisão para identificar lesões valvulares, as decisões diagnósticas e até mesmo terapêuticas às vezes dependem inteiramente de um EC, como o ECO. Apesar de algumas desvantagens, o ECO é atualmente um método de diagnóstico por imagem não invasivo muito valioso para avaliar anormalidades anatômicas e funcionais em todos os tipos de doenças cardíacas.^18 , 19^ Entretanto, nenhum estudo anterior foi desenhado para testar o impacto da realização de EF após a disponibilidade de dados sobre o cenário clínico (anamnese) e um EC como o ECO. Nosso estudo reforça que o EF, uma ferramenta de baixo custo praticamente acessível a todos em condições como doenças valvulares cardíacas, deve ser realizado adequadamente antes do ECO e combinado à avaliação por ECO como um processo de diagnóstico passo a passo.^20^

Os achados do nosso estudo são um tanto discrepantes em relação aos de Hatala et al., que descreveram o impacto clínico de diferentes cenários simulados na interpretação do eletrocardiograma e mostraram que o fornecimento de uma anamnese correta melhorou a precisão em 4% a 12% em comparação com a ausência de tais informações, um efeito mais acentuado para os indivíduos menos treinados (estudantes de graduação).^16^ Em contraste, nossos dados sugerem que os alunos não pareciam influenciados por informações da história clínica para contestar um laudo de ECO. Mesmo entre os alunos que classificaram seu desempenho durante o EF como “bom” e se autoavaliaram como “autoconfiantes” a respeito do diagnóstico clínico, quando confrontados com um laudo clínico ECO Discordante, ainda confiaram na conclusão do laudo do ECO para reclassificar a gravidade da disfunção valvular, indicar tratamento e alterar o diagnóstico clínico. Essa confiança excessiva dos alunos em um laudo de ECO pode estar relacionada à imaturidade e à falta de experiência clínica. Khan et al., estudando a TDC para diagnóstico em um cenário cirúrgico não cardíaco, quantificaram uma associação entre um maior nível de treinamento (especialização) e uma menor taxa de erro diagnóstico.^21^ Nosso estudo não avaliou diferenças no nível de treinamento de graduação e precisão diagnóstica correspondente, nem fez comparações com o desempenho de cardiologistas especialistas. Entretanto, a prática clínica sugere que uma confiança ilimitada em um EC, mesmo ao apresentar características clínicas conflitantes, é mais comum do que o esperado na avaliação de doenças valvulares. Conforme já afirmado nas descobertas de Graber et al. sobre erros de diagnóstico em medicina interna, esse comportamento pode refletir algumas das falhas mais comuns no raciocínio clínico: fechamento prematuro de hipóteses e geração incorreta de contexto.^22^ Graber et al. afirmou que essas armadilhas não estavam necessariamente relacionadas ao conhecimento médico insuficiente, mas ao raciocínio crítico falho e à interpretação inadequada do contexto. Isso levanta preocupações sobre como esse assunto tem sido abordado no currículo das faculdades de medicina, diante do fato de que os alunos ainda estão desenvolvendo habilidades de raciocínio e podem não ser adequadamente encorajados a revisar informações adicionais em cada contexto, aumentando assim as possibilidades diferenciais de diagnóstico.^22^

É reconfortante que até mesmo estudantes de graduação treinados apenas com base em um currículo primário de cardiologia possam identificar corretamente as disfunções valvulares cardíacas mais prevalentes como RM, RA e EA.^4^ Conforme esperado, identificar e caracterizar os sinais de EM no EF foi a tarefa mais desafiadora. A dificuldade na ausculta^14^ da EM pode ser explicada em parte pelas características sonoras complexas do tom inerentemente baixo do sopro diastólico estrondoso (o ouvido humano é muito mais propenso a detectar sons de frequência mais alta). No entanto, a dificuldade também pode ser devido à menor exposição dos alunos aos pacientes com EM durante o curso de medicina. Essa interpretação é consistente com as características epidemiológicas da prevalência da doença valvular em várias regiões. EA e RM apresentam prevalência crescente, contrastando com a redução da prevalência de EM nas últimas décadas. Um relato de Patel et al.^8^ na Índia descreve maior precisão na identificação de sopros de EM associada à maior familiaridade dos médicos locais com esta doença.

O número relativamente alto de ECs solicitados pelos participantes, mesmo quando poderiam realizar um EF, pode ser explicado pela necessidade de maior consideração da relação custo-benefício. Entretanto, esse padrão de uso indiscriminado de EC reflete a prática clínica em muitos cenários, mesmo entre médicos e cardiologistas experientes.^3^ Isso é lamentável, pois a prevalência de algumas doenças valvulares, como EA degenerativa e RM, está aumentando devido ao envelhecimento da população. Por outro lado, a detecção de disfunção valvular cardíaca é, portanto, um dos desafios atualmente não solucionados no tratamento das doenças valvulares cardíacas em Cardiologia, apesar da aplicação extensiva de diretrizes recentes.^23^

Como a indicação terapêutica para intervenção valvular depende em grande parte do laudo do ECO, uma conclusão importante do nosso estudo é que a complexidade da prática clínica garante um equilíbrio cuidadoso entre os achados do EF e os resultados de estudos complementares, evitando subutilização ou confiança excessiva, respectivamente. Para continuar alcançando esses bons resultados na prática clínica, o currículo de medicina deve persistentemente transmitir habilidades de realização de EF cardíaco com toda a sua complexidade, confiando que os alunos de graduação conseguirão adquirir esse aprendizado com boa qualidade.

### Limitações

Nosso estudo contou com diversas limitações, uma delas foi a ausência de comparação entre o desempenho de estudantes de graduação e especialistas em cardiologia na TDC. Embora tivéssemos planejado incluir esse subgrupo, um número insuficiente de especialistas em cardiologia respondeu ao nosso convite para participar. Os alunos da graduação que aceitaram, podem representar um viés populacional de maior interesse em Cardiologia. Outro ponto interessante seria comparar os resultados com uma população de médicos generalistas.

Outra limitação do estudo foi expor os participantes a uma proporção arbitrária de 50% de laudos discordantes de ECO. Os autores reconhecem que essa proporção pode não ser consistente com a realidade da prática clínica e que a discordância dos laudos de ECO em vários cenários clínicos de doenças valvulares ainda não é amplamente conhecida. Além disso, o alto número de EC solicitados pelos participantes, mesmo com realização de EF, pode ter sido influenciado pela possibilidade de escolha de EC sem qualquer limitação de número, custos ou disponibilidade no desenho deste estudo.

## Conclusões

Em vários cenários de disfunção valvular cardíaca, a realização de EF por estudantes de medicina aumentou significativamente a precisão e a confiança do diagnóstico, incluindo a avaliação da gravidade da disfunção valvular, mesmo quando esse aspecto foi avaliado com ECO. Além disso, a qualidade do EF realizado por estudantes de medicina foi boa para reconhecer a presença e a classificação da gravidade da EA e das disfunções aórtica e mitral regurgitantes. Ainda assim, foi documentada uma precisão reduzida no reconhecimento de sinais de EM.
