# Supplementary material for: Como o Exame Físico Cardiovascular Impacta a Tomada de Decisão Clínica em Vários Cenários de Doenças Valvulares Cardíacas
Source: Arq Bras Cardiol. 2025 Mar 6;122(2):e20240272. [Article in Portuguese] doi: 10.36660/abc.20240272 (PMC12087636; doi:10.36660/abc.20240272)
Supplement: Supplementary file 1 [file 0066-782X-abc-122-2-e20240272-suppl01.pdf]

APPENDIX 1: CLINICAL CASE SCENARIOS

Case 1: Mitral Regurgitation

An adult male patient in their 60s, was referred because of dyspnea in moderate physical activities, such as walking through 4 blocks or on slightly level ground. He also complained about orthopnea in the last few weeks. He denies nocturnal paroxysmal dyspnea, edema, syncope, and other cardiovascular symptoms. Symptoms have been occurring for 4 months.

He refers to arterial hypertension in treatment with hydrochlorothiazide. Mentions that a cardiac murmur was identified last year, before symptoms started, during a routine appointment.

In the physical evaluation of this clinical case, you may consider:
Heart rate 70 bpm.
Blood Pressure= 160x94 mmHg

Case 2: Aortic Regurgitation

An adult male patient in their 50s was referred because of dyspnea in moderate physical effort, such as walking more quickly on flat ground, for about a year, worsening to discomfort in small efforts since a month ago. He also reports orthopnea and nocturnal paroxysmal dyspnea in the past weeks and denies having edema.
He has a family history of coronary artery disease, and his father had a heart attack in their 70s. He is a smoker. He also states that a cardiac murmur was identified 5 years ago in a routine appointment, being instructed to continue annual follow-up.

In the physical evaluation of this clinical case, you may consider:
Heart rate 70 bpm.
Blood Pressure= 140 x 86 mmHg

Case 3: Aortic Stenosis

An adult male patient, in their 70s, was referred because of typical chest pain, induced by moderate physical activities, for about 2 months. The pain is severe, with no irradiation, and leads to interrupting his activities, with relief after rest. In one episode, while carrying a bag of cement, there was syncope, with rapid spontaneous consciousness recovery and no trauma. He also refers to dyspnea while performing the same activities that trigger the pain. He denies orthopnea and nocturnal paroxysmal dyspnea, oedema or palpitations.
Medical history of dyslipidemia in treatment and prostate disease.

He refers that a cardiac murmur was identified in a routine appointment a year ago, while asymptomatic, being instructed to continue annual follow-up.

In the physical evaluation of this clinical case, you may consider:
Heart rate 70 bpm. Blood Pressure= 136 x 70 mmHg

Case 4: Mitral Stenosis

A female patient in their 50s, complains about episodic palpitations, more often while performing moderate physical activities, such as heavy housekeeping activities. These events persist until rest and are not followed by chest pain or blurred vision. Episodes of palpitation are concomitant with dyspnea while performing the same physical activities. She denies orthopnea, nocturnal paroxysmal dyspnea, and edema. She also denies bleeding, infectious symptomatology, or weight changes.
She has a medical history of smoking and asthma in childhood.

A cardiac murmur was identified in a previous appointment, being instructed to continue the annual follow-up.

In the physical evaluation of this clinical case, you may consider:
Heart rate 70 bpm. Blood
Pressure= 110 x 68 mmHg
