# Supplementary material for: Como o Exame Físico Cardiovascular Impacta a Tomada de Decisão Clínica em Vários Cenários de Doenças Valvulares Cardíacas
Source: Arq Bras Cardiol. 2025 Mar 6;122(2):e20240272. [Article in Portuguese] doi: 10.36660/abc.20240272 (PMC12087636; doi:10.36660/abc.20240272)
Supplement: Supplementary file 2 [file 0066-782X-abc-122-2-e20240272-suppl02.pdf]

APPENDIX 2: ECHOCARDIOGRAPHY REPORTS

ECHOCARDIOGRAPHY REPORT SUMMARY: CASE 1 - Discordant

TRANSTHORACIC ECHOCARDIOGRAPHY

Examination of regular technical quality, carried out using the highest development equipment.

Patient in sinus rhythm.

Cavity dimensions and LV function:

Parameter	Value	Normality
LA dimension (mm)	54	< 40
LA volume index (ml/m^2)	44	< 34
LV end-diastolic diameter (mm)	62	<56
LV end-systolic diameter (mm)	48	< 45
LV mass index (g/m^2)	140	<115 (male)
RV basal diameter (mm)	44	42
Ejection fraction (2D) (%)	68	>50

LA= left atrial; LV= left ventricular; RV= right ventricular; 2D= two-dimensional or Simpson method.

No evidence of LV segmental wall-motion abnormalities.

Valve quantification parameters:

Thickened mitral valve, small failure of coaptation of leaflets.

Mild mitral regurgitation.

Conclusion:

Marked dilation of the left atrium and moderate dilation of the left ventricle

Preserved global and segmental LV systolic function.

Mild mitral regurgitation.

ECHOCARDIOGRAPHY REPORT SUMMARY: CASE 1 - Concordant

TRANSTHORACIC ECHOCARDIOGRAPHY

Exam of good technical quality, carried out using highest development equipment.

Patient in sinus rhythm.

Cavity dimensions and LV function:

Parameter	Value	Normality
LA dimension (mm)	54	< 40
LA volume index (ml/m^2)	44	< 34
LV end-diastolic diameter (mm)	62	<56
LV end-systolic diameter (mm)	48	< 45
LV mass index (g/m^2)	140	<115 (male)
RV basal diameter (mm)	44	42
Ejection fraction (2D) (%)	68	>50

LA= left atrial; LV= left ventricular; RV= right ventricular; 2D= two-dimensional or Simpson method.

No evidence of LV segmental wall-motion abnormalities.

Valve quantification parameters:

Thickened mitral valve, with significant failure of coaptation of leaflets.

Marked mitral regurgitation, with vena contracta 7mm, jet area/LA area ratio 44%, with systolic reflux in pulmonary veins, and effective regurgitant orifice of 40mm2

Pulmonary systolic pressure estimated by tricuspid regurgitation of 60 mmHg (Normal limits of up to 35 mmHg)

Conclusion:

Marked dilation of the left atrium and moderate dilation of the left ventricle

Preserved global and segmental LV systolic function.

Severe mitral regurgitation.

ECHOCARDIOGRAPHY REPORT SUMMARY: CASE 2 - Discordant

TRANSTHORACIC ECHOCARDIOGRAPHY

Examination of regular technical quality, carried out using highest development equipment. Patient in sinus rhythm.

Cavity dimensions and LV function:

Parameter	Value	Normality
LA dimension (mm)	54	< 40
LA volume index (ml/m^2)	44	< 34
LV end-diastolic diameter (mm)	62	<56
LV end-systolic diameter (mm)	48	< 45
LV mass index (g/m^2)	140	<115 (male)
RV basal diameter (mm)	44	42
Ejection fraction (2D) (%)	68	>50

LA= left atrial; LV= left ventricular; RV= right ventricular; 2D= two-dimensional or Simpson method.

No evidence of LV segmental wall-motion abnormalities.

Valve quantification parameters:
Aortic valve with slight thickening.
Mild aortic regurgitation.

Conclusion:
Marked dilation of the left atrium and moderate dilation of the left ventricle
Preserved global and segmental LV systolic function.
Mild aortic regurgitation.

ECHOCARDIOGRAPHY REPORT SUMMARY: CASE 2 - Concordant

TRANSTHORACIC ECHOCARDIOGRAPHY

Exam of good technical quality, carried out using the highest development equipment.

Patient in sinus rhythm.

Cavity dimensions and LV function:

Parameter	Value	Normality
LA dimension (mm)	54	< 40
LA volume index (ml/m^2)	44	< 34
LV end-diastolic diameter (mm)	62	<56
LV end-systolic diameter (mm)	48	< 45
LV mass index (g/m^2)	140	<115 (male)
RV basal diameter (mm)	44	42
Ejection fraction (2D) (%)	68	>50

LA= left atrial; LV= left ventricular; RV= right ventricular; 2D= two-dimensional or Simpson method.

No evidence of LV segmental wall-motion abnormalities.

Valve quantification parameters:

Thickened aortic valve, with significant failure of coaptation of leaflets.

Marked regurgitation, with 6mm vena contracta, with diastolic reflux in the abdominal aorta.

Pulmonary systolic pressure estimated by tricuspid regurgitation of 50 mmHg (Normal limits until 35 mmHg)

Conclusion:

Marked dilation of the left atrium and moderate dilation of the left ventricle

Preserved global and segmental LV systolic function

Severe aortic regurgitation.

ECHOCARDIOGRAPHY REPORT SUMMARY: CASE 3 - Discordant

TRANSTHORACIC ECHOCARDIOGRAPHY

Examination of regular technical quality, carried out using highest development equipment. Patient in sinus rhythm.

Cavity dimensions and LV function:

Parameter	Value	Normality
LA dimension (mm)	39	< 40
LA volume index (ml/m^2)	32	< 34
LV end-diastolic diameter (mm)	50	<56
LV end-systolic diameter (mm)	35	< 45
LV mass index (g/m^2)	106	<115
RV basal diameter (mm)	41	42
Ejection fraction (2D) (%)	58	>50

LA= left atrial; LV= left ventricular; RV= right ventricular; 2D= two-dimensional or Simpson method.

No evidence of LV segmental wall-motion abnormalities.

Valve quantification parameters:

Aortic valve difficult to visualize, slightly calcified and with only a slight reduction in leaflet opening.

Maximum speed= 220 cm/s.

Peak gradient = 12 mmHg

Conclusion:

Normal sized cardiac chambers.

Preserved global and segmental LV systolic function

Mild aortic valve calcification, without significant gradient.

ECHOCARDIOGRAPHY REPORT SUMMARY: CASE 3 - Concordant

TRANSTHORACIC ECHOCARDIOGRAPHY

Examination of good technical quality, carried out using highest development equipment.

Patient in sinus rhythm.

Cavity dimensions and LV function:

Parameter	Value	Normality
LA dimension (mm)	49	< 40
LA volume index (ml/m^2)	40	< 34
LV end-diastolic diameter (mm)	50	<56
LV end-systolic diameter (mm)	35	< 45
LV mass index (g/m^2)	128	<115
RV basal diameter (mm)	41	42
Ejection fraction (2D)(%)	58	>50

LA= left atrial; LV= left ventricular; RV= right ventricular; 2D= two-dimensional or Simpson method.

No evidence of LV segmental wall-motion abnormalities.

Valve quantification parameters:

Calcified aortic valve with significant reduction in leaflet opening.

AVA= 0.8 cm^2

AVA_i=0.47 cm^2

Maximum speed= 445 cm/s.

Peak gradient = 80 mmHg

Average gradient= 45 mmHg

Conclusion:

Moderate LA dilatation.

LV concentric hypertrophy.

Severe aortic stenosis.

Preserved global and segmental LV systolic function

ECHOCARDIOGRAPHY REPORT SUMMARY: CASE 4 - Discordant

TRANSTHORACIC ECHOCARDIOGRAPHY

Examination of regular technical quality, carried out using highest development equipment. Patient in sinus rhythm.

Cavity dimensions and VE function:

Parameter	Value	Normality
LA dimension (mm)	54 mm	< 40
LA volume index (ml/m^2)	44 ml/m^2	< 34
LV end-diastolic diameter (mm)	48 mm	<56
LV end-systolic diameter (mm)	32 mm	< 45
LV mass index (g/m^2)	78 g/m^2	<95 (five)
RV basal diameter (mm)	44 mm	42
Ejection fraction (2D) (%)	60 %	>50

LA= left atrial; LV= left ventricular; RV= right ventricular; 2D= two-dimensional or Simpson method.

No evidence of LV segmental wall-motion abnormalities.

Valve quantification parameters:

Mitral valve with thickened leaflets, dome-shaped slightly opening reduction, without significant regurgitation.

Mitral valve area difficult to estimate due to difficult aligniment of the jet.

Average gradient= 4 mmHg

It was not possible to estimate systolic pressure in the pulmonary artery.

Conclusion:

Marked dilation of the left atrium.

Preserved global and segmental LV systolic function.

Mitral valve thickened, with only slightly reduced oppening.

ECHOCARDIOGRAPHY REPORT SUMMARY: CASE 4 - Concordant

TRANSTHORACIC ECHOCARDIOGRAPHY

Exam of good technical quality, carried out usinghighest development equipment.

Patient in sinus rhythm.

Cavity dimensions and VE function:

Parameter	Value	Normality
LA dimension (mm)	54 mm	< 40
LA volume index (ml/m^2)	44 ml/m^2	< 34
LV end-diastolic diameter (mm)	48 mm	<56
LV end-systolic diameter (mm)	32 mm	< 45
LV mass index (g/m^2)	78 g/m^2	<95 (five)
RV basal diameter (mm)	44 mm	42
Ejection fraction (2D) (%)	60 %	>50

LA= left atrial; LV= left ventricular; RV= right ventricular; 2D= two-dimensional or Simpson method.

No evidence of LV segmental wall-motion abnormalities.

<u>Valve quantification parameters:</u>

Mitral valve with thickened leaflets, dome-shaped reduced oppening, without significant regurgitation.

Mitral valve area estimated by PHT= 0.9 cm^2 and measured at 0.8 cm^2 by two-dimensional imaging.

Average gradient= 13 mmHg

Williams-Block score of 7

Pulmonary systolic pressure estimated by tricuspid regurgitation of 44 mmHg (Normal limits up to 35 mmHg)

<u>Conclusion:</u>

Marked dilation of the left atrium.

Preserved global and segmental LV systolic performance.

Severe mitral stenosis.
