# Supplementary material for: Como o Exame Físico Cardiovascular Impacta a Tomada de Decisão Clínica em Vários Cenários de Doenças Valvulares Cardíacas
Source: Arq Bras Cardiol. 2025 Mar 6;122(2):e20240272. [Article in Portuguese] doi: 10.36660/abc.20240272 (PMC12087636; doi:10.36660/abc.20240272)
Supplement: Supplementary file 3 [file 0066-782X-abc-122-2-e20240272-suppl03.pdf]

Appendix 3: Digital Forms

Form 1: Case Assessment without Physical Examination

- **General information**
  - Candidate Number:
  - Exam Date:

- **Interpretation and conduct of Pre-Physical Examination**
  - Randomized case:
    - Case 1
    - Case 2
    - Case 3
    - Case 4

  - 1) Based on the information provided in the clinical case, how likely do you consider that "Valve pathology" is the diagnosis of this clinical syndrome?
    - 1 - unlikely
    - 2
    - 3
    - 4
    - 5 - very likely

  - 2) If you considered "Valve pathology" a likely diagnosis, how would you classify its severity?
    - Mild
    - Severe
    - Not applicable

  - 3) With this information, how confident are you when giving this diagnosis?
    - 0 - Very unsure
    - 1
    - 2
    - 3
    - 4
    - 5 - Very confident

  - 4) Consider that you will not perform the Physical Examination on this patient. Based exclusively on the Anamnesis (clinical story), which test(s) would you request to investigate this patient?
    - Electrocardiogram
    - Chest x-ray
    - Echocardiogram
    - Cardiac catheterization
    - Pro-BNP
      Troponin

- **Interpretation and Management after Echocardiography Reports**
    - 5) Based on the test report presented, what is your diagnostic hypothesis?
        - Absence of significant valvular heart disease
        - Aortic stenosis
        - Mitral Stenosis
        - Aortic insufficiency
        - Mitral insufficiency
        - There is not enough data to diagnose a specific valve disease
    - 6) If you have chosen a valve disease, how would you classify its severity?
        - Mild
        - Severe
        - Not applicable
    - 7) How confident are you in this diagnosis?
        - 0 - Very unsure
        - 1
        - 2
        - 3
        - 4
        - 5 - Very confident
        -
    - 8) What would be your next clinical decision given this information?
        - Repeat one of the previously requested tests
        - Observation without the need for treatment
        - Suggest pharmacological treatment
        - Suggest surgical or percutaneous treatment
        - I need to see the results of additional tests first

Form 2: Case Assessment with Physical Examination

- **General information**
    - Candidate Number
    - Randomized case for EF:
        - Case 1
        - Case 2
        - Case 3
        - Case 4

- **Interpretation and Clinical Decisions after Physical Examination**
  - 9) Based now on the clinical case story and the physical examination you have just performed, what is your diagnostic hypothesis?
    - Absence of significant valvular heart disease
    - Aortic stenosis
    - Mitral Stenosis
    - Aortic insufficiency
    - Mitral insufficiency
    - There is not enough data to diagnose specific valvulopathy
  - 10) If you have chosen a valve disease, how would you classify its severity?
    - Mild
    - Severe
    - Not applicable
  - 11) How would you evaluate your performance in the physical examination?
    - Incomplete
    - Partially incomplete
    - Neutral
    - Partially complete
    - Complete
  - 12) How confident are you in this diagnosis?
    - 0 - Very unsure
    - 1
    - 2
    - 3
    - 4
    - 5 - Very confident
  - 13) Based on the clinical history and your physical examination, which test(s) would you request to investigate this patient? You can choose more than one.
    - Electrocardiogram
    - Chest x-ray
    - Echocardiogram
    - Cardiac catheterization
    - Pro-BNP
    - Troponin
  - 14) Based on the clinical history and your physical examination, what would be your next clinical decision given this information?
    - Observation without the need for treatment
    - Pharmacological treatment
    - Surgical or percutaneous treatment
    - I need to see the results of additional tests first

- **Interpretation and Management After Echocardiography Result**
  - 15) Based on the test results presented, what is your diagnostic hypothesis? (It may be the same as the previous one).
    - Absence of significant valvular heart disease
    - Aortic stenosis
    - Mitral Stenosis
    - Aortic insufficiency
    - Mitral insufficiency

  - 16) If you have chosen a valve disease, how would you classify its severity?
    - Mild
    - Severe
    - Not applicable

  - 17) How confident are you in this diagnosis?
    - 0 - Very unsure
    - 1
    - 2
    - 3
    - 4
    - 5 - Very Confident

  - 18) What would be your next clinical decision given this information?
    - Repeat one of the previously requested exams
    - Observation without the need for treatment
    - Suggest Pharmacological treatment
    - Suggest surgical or percutaneous treatment
    - I need to see the results of additional tests first

Form 3 - OSCE: Physical Examination Assessment

- **General information**
  - Candidate Number:
  - Data
  - Appraiser Name:
    - Minna Romano
    - Natan Viola
    - Isabella Teixeira
    - Vinícius Lima
  - Year of Study
    - Fourth Year
    - Fifth year
    - Sixth year
    - Cardiology resident
    - Cardiologist
  - Group:
    - A
    - B
  - First Case randomized (without PE)
    - Case 1
    - Case 2
    - Case 3
    - Case 4
  - ECHO 1 received:
    - Concordant
    - Discordant
  - Second Case randomized (without PE)
    - Case 1
    - Case 2
    - Case 3
    - Case 4
  - ECO 2 received:
    - Concordant
    - Discordant
  - Third Case randomized (With PE)
    - Case 1
    - Case 2
    - Case 3
    - Case 4
  - ECO 3 received:
    - Concordant
    - Discordant
  - Fourth Case randomized (with PE)
    - Case 1
    - Case 2
    - Case 3
    - Case 4
  - ECO 4 received:
    - Concordant
    - Discordant

- **Description of EF Cardiovascular**
  - Inspection of the precordium: Ictus
    - Normally positioned
    - Left deviation
    - Not visible
  - Palpation of the precordium:
    - Normally positioned stroke
    - Left deviation
    - Non Palpable Ictus
  - Inspection of the precordium
    - Visible parasternal pulsation
    - Parasternal impulse not visible
  - Study of S2:
    - Normal
    - Physiological Unfolding
    - Did not describe
  - Additional rumbles or noises
    - S3
    - S4
    - None
  - Additional sounds
    - Aortic ejection click
    - Mitral opening click
    - None
  - Palpation of the carotid pulses:
    - Normal
    - Wide
    - Water Hammer/Corrigan
    - *Parvus et tardus*
  - Murmur:
    - Systolic
    - Diastolic

Completed if: Systolic Murmur

- Murmur grading:
  - 1
  - 2
  - 3
  - 4
  - 5
  - 6
- Timing Description:
  - Holosystolic
  - Midsystolic
  - Early systolic
  - I can't say
- Shape Description:
  - Crescendo-decrescendo
  - Plateau/Uniform
  - I didn't know how to characterize
- Listening areas:
  - Aortic focus
  - Pulmonary focus
  - Tricuspid focus
  - Mitral focus
- Irradiation:
  - Wishbone and Carotids
  - Subclavicular
  - Left sternal border
  - Left axillary region
  - Does not irradiate

Completed if: Diastolic murmur

- Murmur grading:
  - 1
  - 2
  - 3
  - 4
- Timing Description:
  - Early diastolic
  - Mid diastolic
  - I can't say
- Shape Description:
  - Uniform
  - Decrescendo
  - I can't say
- Listening Posts:
  - Aortic focus
  - Pulmonary focus
  - Tricuspid focus
  - Mitral focus
- Irradiation:
  - Wishbone and Carotids
  - Subclavicular
  - Left sternal border
  - Left axillary region
  - Does not irradiate
