# Supplementary material for: Como o Exame Físico Cardiovascular Impacta a Tomada de Decisão Clínica em Vários Cenários de Doenças Valvulares Cardíacas
Source: Arq Bras Cardiol. 2025 Mar 6;122(2):e20240272. [Article in Portuguese] doi: 10.36660/abc.20240272 (PMC12087636; doi:10.36660/abc.20240272)
Supplement: Translation

# Translation: How Cardiovascular Physical Examination Impacts Clinical Decision-Making in Various Scenarios of Cardiac Valvular Diseases

**DOI:** 10.36660/abc.20240272i

**Authors:** Isabella Silveira Teixeira, Vinícius Lima Borges, Natan Viola, Henrique Turin Moreira, Antonio Pazin, André Schmidt, José Antônio Marin-Neto, Minna Moreira Dias Romano

**Affiliations:** 1 Brazil Faculdade de Medicina de Ribeirão Preto da Universidade de São Paulo, Ribeirão Preto, SP – Brazil

**Keywords:** Physical Examination, Echocardiography, Clinical Decision-Making, Heart Valve Diseases

## Abstract

**Background:**

Complementary diagnostic examinations have replaced the importance of Physical Examination (PE) in Clinical Decision-making (CDM).

**Objective:**

To evaluate the impact of cardiovascular PE in scenarios of cardiac valvular diseases.

**Methods:**

An interventional study with volunteers’ patients having or not having the opportunity to perform PE in scenarios of valvopathies. The PE was simulated in a high-fidelity cardiopulmonary simulator. Volunteers received questions about CDM before and after receiving an ECHO Concordant or ECHO Discordant report. Cohen´s kappa coefficient and square contingency tables compared diagnostic accuracy. ANOVA tests compared the number of requested tests; the significance level was set as p < 0.05.

**Results:**

Sixty volunteers performed 239 clinical observations in 4 valvular dysfunctions. The diagnostic accuracy of valvular dysfunction was good (kappa = 0.935, p < 0.001). After receiving ECHO reports, the accuracy was worse without PE (p = 0.0047). The confidence level in diagnosis was 28.18% higher when PE was performed (p < 0.01). However, after receiving ECHO reports, diagnostic confidence levels were only 4% higher in the group with PE (p = 0.03). There were significantly more CATE requests when PE was not performed (p = 0.0326). The indication of valvular intervention was not related to having or not having the opportunity to perform PE (79 with PE vs 78 without PE, p = 0.0607), but was influenced by ECHO Concordant vs ECHO Discordant reports (p < 0.001).

**Conclusions:**

Performing PE enhanced diagnosis confidence in valvulopathy scenarios and correct dysfunction grading. Treatment decisions were more based on ECHO reports than on PE when they were discordant, and requests for CATE increased when volunteers had no chance to perform PE.

## Key Messages

1. Medical clinical practice has lost confidence in Physical Examination (PE) compared to complementary diagnostic examinations (CE). More importance has been deposited on Echocardiography (ECHO) in cardiac valvular dysfunction scenarios. No studies have measured the impact of cardiac PE in Clinical Decision-making (CDM).

2. Discrepancies between PE findings and ECHO reports are common and may impact CDM in different forms.

3. PE has proven essential to enhancing diagnosis confidence and grading of dysfunction in cardiac valvular dysfunctions. ECHO still plays a significant importance in CDM, even when discordant from physical examination.

4. The number of solicitations of an invasive test such as Cardiac Catheterization (CATE) is influenced by PE. The number of orderings was higher when volunteers did not perform a PE.

## Introduction

Following a centuries-honored reputation within the medical practice, in the last decades, the importance of Physical Examination (PE) for Clinical Decision-making (CDM) lost most of its appeal, and a neat trend for its replacement by various complementary diagnostic examinations (CE) ensued.^1^In Cardiology, a genuine concern has arisen about the actual importance of PE since the development of so many different imaging diagnosis tests, among which the most available was transthoracic echocardiography (ECHO). However, using a CE test not preceded by a proper PE may increase costs and time to diagnose and potentially cause severe distortions, including prognostic mistakes.^2^Another predictable consequence of the trend is that practitioners may have lost the clinical ability to detect cardiac diseases such as valvulopathy,^1 , 3^thus severely impacting professionals’ confidence in cardiovascular PE. Also, although the teaching of cardiovascular PE has been improved with the use of high-performance simulators, there is clear evidence that educative skills to teach cardiac PE have been weakened in the curriculum of many medical courses.^3 - 7^

There is substantial knowledge about the value of cardiovascular PE in detecting abnormalities such as valvular diseases and elevation of cardiac pressures, arrhythmias, chamber dilatation, and systolic and diastolic dysfunction.^1 , 8^Most published studies compared the agreement between cardiac physical signs and other diagnostic methods, such as ECHO^1^or cardiac catheterization (CATE), through measurements of intracardiac pressures and other indices of abnormal structure and function of the heart. However, no prior study had tested the role of PE in CDM, particularly when considering the impact of a more sophisticated yet accessible CE such as ECHO.^9^

CDM involves technical abilities and complex and “difficult-to-measure” factors such as individual analytic or “pattern-recognition” types of cognitive thinking.^10 , 11^Previous studies, such as those by Sibbald et al., established the role of anamnesis as a tool to enhance diagnosis in simulated valvulopathies, where PE was structured and simulated in high-fidelity cardiopulmonary systems.^12^Hampton et al. evaluated the relative importance of anamnesis added to PE and laboratory tests in CDM in some general clinical scenarios but not specifically in valvopathies. In a group of 80 patients with cardiac diseases, anamnesis positively influenced the achievement of diagnosis in 80% of the studied situations, but only in a few proportions of patients; the diagnosis was enhanced when PE was added to anamnesis.^13^Although PE may also have had a significant role in prognostic and therapeutic decisions, these aspects were not thoroughly evaluated in those studies.

We sought to evaluate the cardiovascular PE impact in CDM of undergraduate medical students in some scenarios of severe cardiac valvular diseases, simulated with a high-fidelity cardiopulmonary simulator.^10 , 14^

## Methods

The study was designed as interventional, in which the intervention variable was the cardiovascular PE, and the dependent variables (outcomes) were answers reflecting CDM. It was approved by the institutional Research Ethics Committee (process number HC-FMRP-5.046.039).

### Population

Undergraduate medical students from the University of São Paulo Ribeirão Preto Medical School, running in the 4^th^, 5^th^, or 6^th^graduate year academic course, were invited to participate. The convenience sample size was based on 200 available students at the time of study. Students received written invitations and instructions from institutional email and social media groups. Participants should be students previously approved in the cardiovascular discipline of their clinical course of the 4^th^academic year, and participation was completely voluntary. All the students who participated provided written consent.

### Simulation

The PE was performed in a high-fidelity cardiopulmonary simulator (Harvey^@^, Gordon Center for Research in Medical Education, University of Miami, FL, USA), capable of reproducing normal and abnormal physical signs of several cardiovascular conditions. Although the entire group of students had previously acquired experience with the Harvey simulator during the cardiovascular course, they all received additional standardized training in using the Harvey simulator with various example simulated conditions after volunteering to participate.

### Design

A simulation of four valvular disease scenarios (Mitral Stenosis (MS), Mitral Regurgitation (MR), Aortic Stenosis (AS), and Aortic Regurgitation (AR)) was designed by two experienced cardiologists. Each scenario started with a brief clinical case synopsis ( Appendix 1 ), which provided information about a fictional patient (age, gender, short characterization symptoms such as dyspnea, angina, edema, palpitation, syncope, and mention of an individual antecedent of a non-specified cardiac murmur). After reading the anamnesis and synopsis of each scenario, volunteers were randomly divided into groups with or without the chance to perform cardiovascular PE in the simulator and then received a written ECHO report. It was a simple randomization process based on computer-generated tables within the statistical software. All the valvular disease scenarios were planned as typical, with apparent symptoms and cardiovascular PE simulation, which included signs of high severity.

An experienced echocardiographer developed the ECHO reports. Two different reports were elaborated for each of the valvular scenarios: a concordant report (ECHO Concordant) and a discordant report (ECHO Discordant) ( Appendix 2 ). The concordant report presented evidence of severe valvulopathy concordant with symptoms and PE. It showed measurements such as valvular area < 1 cm^2^in aortic or mitral stenosis, elevated medium transvalvular gradients, and severe aortic or mitral regurgitation quantifications. The discordant report (ECHO Discordant) had inconsistent information (valvular area or gradients not provided, missing anatomic information, or no disclosure of regurgitation severity, which was only classified as a mild valvular disease).

Applying scenarios to volunteer participants was conducted as a formative objective structured clinical exam (OSCE) ( Appendix 3 ). Each participant was exposed to the four cases of valvulopathy scenarios, MS, MR, AS, and AR, in random sequence order (Groups A and B) ( Figure 1 ). To randomize the chance to perform PE, the interventional variable, volunteers were randomly sorted to have the opportunity to perform PE in half of the scenarios. After this stage, they received the ECHO report in a randomized chance to be an ECHO Concordant or ECHO Discordant in the simulated clinical case.

**Figure 1:** – Distribution of valvular scenarios in groups with and without the possibility of performing physical examination (PE).

Questions reflecting CDM were applied to volunteers after receiving anamnesis and having or not having the chance to perform cardiovascular PE. In a subsequent step, they received ECHO Concordant or ECHO Discordant reports. The mean duration of OSCE was 20 minutes.

### Outcomes

A CDM was evaluated as answers to questions related to clinical diagnosis, etiology of cardiac valvular diseases, level of confidence in diagnosis, and types of complementary diagnostic tests and treatment decisions ( Appendix 3 ). Questions related to diagnosis were presented after the opportunity not to perform cardiovascular PE and again after reading the ECHO Concordant or ECHO Discordant reports. The questions on diagnosis included whether the “valvular pathology” hypothesis was likely, its severity, the level of confidence in diagnosis, and the selection of CE tests. Questions related to treatment decisions were only presented after reading ECHO reports. At this point, volunteers were asked again about the kind of valvular pathology, its severity, their level of confidence in diagnosis, and the therapeutic decision. By design, half of the volunteers were required to answer CDM questions even for scenarios in which they did not have the opportunity to perform cardiac PE, relying solely on anamnesis and ECHO report information. The confidence level in the diagnosis hypothesis before and after the ECHO interpretation was rated on a 6-point Likert^15^scale and coded as mean +-SD.

### Quality assessment of physical examination

The performance of each volunteer in detecting abnormalities during the PE using the Harvey simulator was evaluated by one of the investigators with an OSCE checklist ( Appendix 3 ), adapted from previous validated cardiovascular PE checklists.^10 , 16^The complete inspection of the precordial region, palpation of right ventricle impulse and cardiac apex, auscultation of heart sounds (S3, S4, diastolic and systolic murmurs, clicks, and opening snaps), and characterization of the design of carotid arterial pulses (if regular, *parvus-tardus* or if a Corrigan´ pulse) were all included in the checklist. The volunteers had ten minutes to complete the PE. Before heading to the questionnaire and ECHO interpretation, they were requested to nominate all the normal or abnormal findings for that simulated patient to the examiner. Volunteers were allowed to re-examine the simulator when in doubt. The presence or absence of findings were coded as binary variables. Each volunteer answered the questionnaire in a digital form on each personal smartphone.

### Data description and statistical analysis

The quality of PE was expressed in percentages of correct answers compared to a template of the Harvey simulator manuals. Correct answers were valued when volunteers nominated signs of PE that were present on the manufacturer´s template. Answers to CDM questions were gathered from all volunteers in a single spreadsheet (Excel). Categorical variables were expressed as percentages and frequencies, and continuous variables were expressed as means and standard deviations. The confidence level in diagnosis pre- and post-ECHO interpretation was compared using the unpaired Student´s T-test as appropriate. The normality of continuous data was tested with the Kolmogorov–Smirnov test. Cohen´s kappa coefficient and square contingency tables were used to compare the outcome of diagnosis accuracy between the four valvular pathologies before and after PE and before and after ECHO reports. One-way ANOVA tests (with no post hoc test) were used to compare the number of diagnostic tests requested by volunteers in different valvular pathological scenarios. The identification of incorrect signs and omitted answers were interpreted as wrong. All statistical analyses were performed using Stata 14.0 software (StataCorp, College Station, TX), and the significance level was set as p < 0.05.

## Results

The study population comprised 60 volunteers who participated in four types of valvular dysfunction scenarios, leading to 239 observations, accounting for one instance of missing data. Half of the volunteers performed PE (resulting in 120 cases), and the other did not have the opportunity to perform PE (119 cases due to a missing one). Twenty-three volunteers were running through the 4^th^year, 23 in the 5^th^year, and 14 volunteers in the 6^th^year of the medical academic curriculum (6 years in our Medical School). The overall quality of cardiac PE is demonstrated in Figure 2 .

**Figure 2:** – Quality of physical examination considering all simulated cases of four cardiac valvular dysfunctions.

Volunteers correctly detected *ictus cordis* in 75% (n = 90) of cases. Right ventricular parasternal impulse was identified in 71% of cases (n = 85). Heart murmurs were correctly identified in more than 76% of cases (n = 91), mainly when there were systolic murmurs. Characteristics of systolic murmurs as irradiation were correctly identified in more than 77% (n = 92) of cases, but the percentage of correct answers was less than 60% (n = 63) for diastolic murmurs. Additional sounds, such as S3 and S4, were recognized in 44 % (n = 53) of cases. When the quality of PE was compared between different valvulopathies ( Figure 3 ), signs of systolic-related murmurs such as AS and MR were more easily recognized than diastolic-related murmurs such as MS and AR. The worse performance of cardiac PE was related to MS murmur identification and characterization.

**Figure 3:** – Quality of PE considering each valvular dysfunction: (A) Mitral Regurgitation, (B) Aortic Stenosis, (C) Aortic Regurgitation, and (D) Mitral Stenosis

Diagnostic ability to identify the kind of valvular dysfunction (if AS, AR, MR, or MS) was good (kappa = 0.935, p < 0.001, confidence interval [0.798-0.939]) even when volunteers received an ECHO-discordant report ( Table 1 ).

**Table 1:** – Agreement between valvular dysfunction diagnosis before and after receiving ECHO reports

Diagnosis pre-ECHO	Diagnóstico pós-ECO				Sum
	1	2	3	4	
1	55	0	1	4	60
2	1	51	1	1	54
3	0	0	54	0	54
4	3	0	0	55	58
**Sum**	59	51	56	60	226

Thirteen volunteers did not answer. Scenarios: 1 = Mitral Regurgitation (MR), 2 = Aortic Regurgitation (AR), 3 = Aortic Stenosis (AS), and 4 = Mitral Stenosis (MS).

Volunteers who had no chance to perform PE were less apt to correctly evaluate the valvular dysfunction grade (the correct answer) after receiving ECHO Concordant or ECHO Discordant reports (the correct answer about severity with PE = 85 vs without PE = 64; p = 0.0047), (kappa = 0.2887, p< 0.001, CI [0.797-0.931]) ( Table 2 ).

**Table 2:** – Agreement between valvular dysfunction grading before and after receiving ECHO reports

Pre ECHO grading	Post ECHO grading			Sum
	1	2	3	
1	40	28	0	68
2	40	109	0	149
3	11	8	3	22
**Sum**	91	140	8	239

The confidence level (Likert scale) in the diagnosis was 28.18% higher in the group of volunteers who performed PE. When confidence in diagnosis was confronted with ECHO reports, which could be ECHO Concordant or ECHO Discordant (the main discordance was about the grading of valvulopathy), diagnosis confidence levels were enhanced by only 4% with PE performance ( Table 3 ).

**Table 3:** – Level of confidence in valvular dysfunction

	Likert Scale	Statistical significance
With PE	4.64	p < 0.01
Without PE	3.62	
With PE after ECHO reports	5.24	p = 0.03
Without PE after ECHO reports	5.03	

Volunteers asked for a median of three complementary diagnostic tests; most of the time, it was an EKG, chest X-ray, or ECHO ( Figure 4 ).

**Figure 4:** – Proportion of complementary exams required by the volunteers for diagnosis.

The total number of required CE diagnostic tests was 650, and 328 were indicated by the group of volunteers who performed PE, while 322 were indicated by the group that did not perform PE. The number of indications of CE tests was similar among the four scenarios of valvopathies (ANOVA p = 0.117 compared to the four groups).

When considering the performance of PE, there was a significantly higher number of CE required by the group without PE for specific scenarios of AR cases (74 with PE vs 103 without PE; p = 0.0009). In contrast, more CE was required by volunteers who performed PE in the scenarios of AS cases (93 with PE vs 69 without PE; p = 0.0083) and ME (84 with PE vs 70 without PE; p = 0.045), and no significant difference observed in MR (77 with PE vs. 80 without PE; p > 0.05). Also, considering all scenarios of valvulopathy dysfunctions, there was a significantly higher number of CATE requested by volunteers who could not perform PE (13 without PE vs 8 with PE, p = 0.0326).

Therapeutic indication of valvular intervention was not influenced by the performance of PE (79 with PE vs 78 without PE, p = 0.0607). However, volunteers who received ECHO Concordant reports chose significantly more times the option of valvular dysfunctions surgical treatment (p < 0.001) as compared to those who received ECHO Discordant reports ( Table 4 ).

**Table 4:** – Number of choices for indication of valvular interventions

	Valvular intervention	Other choice of treatment	Sum
ECHO Concordant	103	17	120
ECHO Discordant	54	66	120
Sum	157	83	240

Valvular intervention: surgical or percutaneous.

## Discussion

The results of this investigation showed that, in a group of medical undergraduate students who already had passed through the cardiovascular discipline (4^th^year of the academic curriculum), the level of confidence in diagnosing valvular dysfunctions and their severity, even after receiving an ECHO report, was enhanced when volunteers had the opportunity to perform PE. Performing PE showed no incremental ability to detect what kind of valvular dysfunction was simulated once the ECHO report was considered. However, volunteers who could perform PE had a better ability not to influence valvular dysfunction grade and to question ECHO reports than the group who had to achieve CDM only with anamnesis and ECHO. Performing PE also did not affect the indication of surgical treatment of any valvular dysfunction. Moreover, receiving an ECHO Concordant or ECHO Discordant report regarding the clinical scenarios significantly influenced the suggested treatment for the specific valvular disease, regardless of whether PE was performed.

Another relevant finding of the study was that, overall, the performance of medical students in PE was good for detecting several signs of valvular diseases. Performance was better in detecting regurgitant valvular signs and AS and worse in detecting signs of MS. Also, except for the request for CATE, there was no increase in the indications of additional CE to make the diagnosis when the PE was not performed. This reflects that all ECHO reports, even the ECHO Discordant report, correctly showed signs of the simulated valvular dysfunction, and the presented discordance was related to the severity of the disease.

Previous studies evaluated the accuracy of single PE signs of valvular dysfunction to identify pathological conditions compared to other instrumented diagnostic tools, such as ECHO.^1 , 17^It is relevant to emphasize that disregarding a simple tool such as PE, which can be performed with good accuracy to identify valvular lesions diagnostic and even therapeutic decisions are sometimes entirely dependent on CE such as ECHO. Despite some pitfalls, ECHO is now a most valuable non-invasive imaging diagnostic method to assess anatomy and functional abnormalities in every kind of heart diseases.^18 , 19^However, no previous study was designed to test the impact of performing PE after data regarding the clinical scenario (anamnesis) and a CE such as ECHO were available. Our study supports that PE, a low-cost tool virtually accessible to everyone in conditions such as cardiac valvular diseases, should be appropriately performed before and added to the ECHO evaluation as a step-by-step diagnosis process.^20^

The findings of our study are somewhat discrepant regarding those of Hatala et al., who described the clinical impact of different simulated scenarios on the electrocardiogram interpretation and showed that the provision of a correct anamnesis improved accuracy by 4% to 12% compared with no such information, an effect more marked for the less trained individuals (undergraduate students).^16^In contrast, our data suggest that students did not seem influenced by clinical history information to contest an ECHO report. Even among students who rated their performance during PE as “good” and self-evaluated themselves as “self-confident” with the clinical diagnosis, when confronted with a clinical ECHO Discordant report, they still relied on the ECHO report´s conclusion to re-classify valvular dysfunction severity, indicate treatment and change the clinical diagnosis. This excessive confidence in an ECHO report by students may be related to students’ immaturity and lack of clinical experience. Khan et al., studying CDM to diagnosis in a non-cardiac surgical scenario, quantified an association between a higher level of training (expertise) and a lower diagnostic error rate.^21^Our study did not assess differences in the undergraduate level of training and correspondent diagnostic accuracy, nor did it compare it to cardiologist specialists’ performance. However, clinical practice suggests that an unlimited trust in a CE, even when presented with conflicting clinical features, is more common than expected in evaluating valvular diseases. As already stated by Graber´s findings on diagnostic errors in intern medicine, this behavior may reflect some of the most common failures in clinical reasoning: premature closure of hypothesis and faulty context generation.^22^Graber et al. asserted that these pitfalls were not necessarily related to insufficient medical knowledge but to flawed critical reasoning and inappropriate context interpretation. That raises concern about how this subject has been approached in the curriculum of medical schools, to face the fact that students are still developing reasoning skills and may not be adequately encouraged to review additional information under each context, thus raising differential possibilities for a diagnosis.^22^

It is reassuring that even undergraduate students trained with only a primary cardiology curriculum can correctly identify the most prevalent cardiac valvular dysfunctions as MR, AR, and AS.^4^As expected, identifying and characterizing the PE signs of MS was the more challenging task. Difficulty in the auscultation^14^of MS can be partly explained by the complex sound features of the inherently low pitch of the rumbling diastolic murmur (the human ear is far more prone to detect higher-frequency sounds). However, the difficulty may also be due to students’ less exposure to MS patients through the medical course. This interpretation is consistent with the epidemiological features of valvular disease prevalence in various regions. AS and MR show increasing prevalence, contrasting with the reduced prevalence of MS in the last decades. A report by Patel et al.^8^in India describes greater accuracy in identifying MS murmurs associated with higher local physicians’ familiarity with this disease.

The relatively high number of CE required by volunteers, even when they could perform PE, can be explained by the need for more consideration of cost-effectiveness. However, this pattern of indiscriminate use of CE reflects clinical practice in many scenarios, even among experienced physicians and cardiologists.^3^This is unfortunate since the prevalence of some valvular diseases, such as degenerative aortic stenosis and mitral regurgitation, is now increasing due to the aging of the population. On the other hand, the still detection of cardiac valvular dysfunction is, therefore, one of the challenges currently unmet in dealing with valvular heart diseases in Cardiology, despite the extensive use of recent guidelines.^23^

Since the therapeutic indication for valvular intervention largely depends on the ECHO report, a key takeaway of our study is that the complexity of clinical practice warrants a careful balance between PE findings and results from complementary studies, avoiding underuse or overreliance, respectively. To keep achieving these good clinical practice outcomes, the medical teaching curriculum must insist on teaching the abilities of cardiac PE with all of its complexity, trusting undergraduate students can respond to this learning with good quality.

### Limitations

Our study had several limitations, one of them being the absence of a comparison between the performance of undergraduate students and cardiology specialists in CDM. Although we had planned to include this subgroup, an insufficient number of cardiology specialists responded to our invitation to participate. Undergraduate volunteers who accepted participation may impose a population bias of better interest in Cardiology. Another interesting point would be comparing results with a generalist doctor’s population.

Another limitation of the study was to expose volunteers to an arbitrary proportion of 50% ECHO discordant reports. The authors acknowledge that this proportion may not be consistent with the reality of clinical practice and that the discordance of ECHO reports in various clinical scenarios of valvular diseases has yet to be generally known. Also, the high number of CE tests requested by volunteers, even with PE performance, could have been influenced by the possibility of choosing CE without any limitation of number, costs, or availability in the design of this study.

## Conclusions

In various scenarios of cardiac valvular dysfunction, performing PE by undergraduate medical students significantly enhanced diagnostic accuracy and confidence, including assessing the severity of valvular dysfunction, even when this aspect had been evaluated with ECHO. In addition, the quality of PE performed by medical students was good for recognizing the presence and severity grading in aortic stenosis and regurgitant aortic and mitral dysfunctions. Still, a reduced accuracy was documented in recognizing signs of mitral stenosis.
